# Computed Tomography Imaging of Solid Tumors Using a Liposomal-Iodine Contrast Agent in Companion Dogs with Naturally Occurring Cancer

**DOI:** 10.1371/journal.pone.0152718

**Published:** 2016-03-31

**Authors:** Ketan B. Ghaghada, Amy F. Sato, Zbigniew A. Starosolski, John Berg, David M. Vail

**Affiliations:** 1 The Singleton Department of Pediatric Radiology, Texas Children’s Hospital, Houston, Texas, United States of America; 2 Department of Clinical Sciences, Tufts Cummings School of Veterinary Medicine, North Grafton, Massachusetts, United States of America; 3 Department of Medical Sciences, School of Veterinary Medicine, University of Wisconsin-Madison, Madison, Wisconsin, United States of America; Brandeis University, UNITED STATES

## Abstract

**Objectives:**

Companion dogs with naturally occurring cancer serve as an important large animal model in translational research because they share strong similarities with human cancers. In this study, we investigated a long circulating liposomal-iodine contrast agent (Liposomal-I) for computed tomography (CT) imaging of solid tumors in companion dogs with naturally occurring cancer.

**Materials and Methods:**

The institutional animal ethics committees approved the study and written informed consent was obtained from all owners. Thirteen dogs (mean age 10.1 years) with a variety of masses including primary and metastatic liver tumors, sarcomas, mammary carcinoma and lung tumors, were enrolled in the study. CT imaging was performed pre-contrast and at 15 minutes and 24 hours after intravenous administration of Liposomal-I (275 mg/kg iodine dose). Conventional contrast-enhanced CT imaging was performed in a subset of dogs, 90 minutes prior to administration of Liposomal-I. Histologic or cytologic diagnosis was obtained for each dog prior to admission into the study.

**Results:**

Liposomal-I resulted in significant (p < 0.05) enhancement and uniform opacification of the vascular compartment. Non-renal, reticulo-endothelial systemic clearance of the contrast agent was demonstrated. Liposomal-I enabled visualization of primary and metastatic liver tumors. Sub-cm sized liver lesions grossly appeared as hypo-enhanced compared to the surrounding normal parenchyma with improved lesion conspicuity in the post-24 hour scan. Large liver tumors (> 1 cm) demonstrated a heterogeneous pattern of intra-tumoral signal with visibly higher signal enhancement at the post-24 hour time point. Extra-hepatic, extra-splenic tumors, including histiocytic sarcoma, anaplastic sarcoma, mammary carcinoma and lung tumors, were visualized with a heterogeneous enhancement pattern in the post-24 hour scan.

**Conclusions:**

The long circulating liposomal-iodine contrast agent enabled prolonged visualization of small and large tumors in companion dogs with naturally occurring cancer. The study warrants future work to assess the sensitivity and specificity of the Liposomal-I agent in various types of naturally occurring canine tumors.

## Introduction

Due to ease of access and low cost, contrast-enhanced CT (CECT) imaging plays an important role in the management of human cancer patients. The contrast agents used in CT imaging are highly water soluble, iodine-based, low molecular weight organic molecules with rapid renal-clearing pharmacokinetics. The short blood half-life of these agents necessitates bolus administration of concentrated solution, synchronized with CT imaging to facilitate optimal opacification within the volume of interest, for acquisition of diagnostic images. The bolus administration combined with the heterogeneous and rapid contrast agent flow dynamics can present challenges in the diagnosis of malignant lesions [1–3]. Studies have shown that CT has lower sensitivity compared to magnetic resonance imaging (MRI) and positron emission tomography (PET) in the diagnosis of liver lesions [3,4]. Additionally, contrast-induced nephropathy remains a concern especially in older and cancer patients with impaired renal function [5,6].

Liposomal-based contrast agents have been investigated for use with a variety of imaging modalities [7–9]. For CT contrast agents, encapsulation of conventional iodine-based molecules within the core interior of the liposomes results in altered bio-distribution and pharmacokinetics [10–12]. Rather than the typical renal clearance, liposomal contrast agents are systemically cleared via uptake by the reticulo-endothelial system (RES). Surface modification with a hydrophilic polymer, such as polyethylene glycol (PEG), increases the blood-residence time of the liposomes [13]. These characteristics make long circulating liposomal-iodine attractive for use as a CT contrast agent because its persistence in the blood pool obviates the need for exact timing of contrast injection during scanning and its non-renal clearance via the RES may decrease nephrotoxicity. Additionally, their ability to extravasate and accumulate in solid tumors, in part due to the enhanced permeability and retention (EPR) effect has enabled efficacy investigations of liposomal contrast agents in cancer imaging, the majority of them conducted in rodent models of human cancers [14].

Companion dogs develop naturally occurring cancers that share strong similarities with human cancers and their inclusion in preclinical modeling studies provides a unique and clinically relevant opportunity in translational research [15,16]. In the context of cancer imaging, their use enables proof-of-concept investigations of novel imaging agents/modalities for naturally occurring tumors. It also allows for investigations of clinically relevant lesion sizes on clinically-equivalent CT scanners.

The purpose of this feasibility study was to perform an investigation of a long circulating liposomal-iodine contrast agent (Liposomal-I) for CT imaging of solid tumors in companion dogs with naturally occurring cancer. More specifically, this study sought to understand the signal enhancement patterns for visualization of primary and metastatic solid tumors in CT scans acquired using the Liposomal-I. A secondary goal of this study was to determine post-contrast temporal changes in CT signal for major vascular structures and target organs.

## Materials and Methods

The use of client owned animals was approved by the Institutional Animal Care and Use Committees of the University of Wisconsin-Madison School of Veterinary Medicine and the Tufts Cummings School of Veterinary Medicine. Written informed consent was also obtained from all owners.

### Contrast Agent

The liposomal-I was prepared as per procedures described previously [17]. Briefly, a lipid mixture (150 mmol/L) consisting of 1,2-dipalmitoyl-sn-glycero-3-phospho- choline (DPPC), cholesterol, and 1,2-distearoyl-sn-glycero-3-phosphoethanolamine-N-[methoxy (polyethylene glycol)-2000] (DSPE- MPEG2000) in a 56:40:4 molar ratio was dissolved in ethanol at ~65°C. The ethanolic lipid solution was hydrated with an iodixanol solution (550 mg I/mL) at ~65°C and then sequentially extruded on a Lipex Thermoline extruder (Northern Lipids, Vancouver, British Columbia, Canada) to size the liposomes to ~140 nm. The resulting solution was diafiltered using a tangential flow filtration module (Spectrum Laboratories, CA) to remove free iodixanol. The size distribution of liposomes in the final formulation was determined by dynamic light scattering (DLS). The iodixanol concentration in the final product was determined using a HPLC method. The product was manufactured as a sterile, ready-to-use suspension. The average liposome size in the final formulation was 135±20 nm and the polydispersity index was below 0.15. The overall iodine concentration was ~110 mg I/mL with more than 95% of iodixanol stably encapsulated within the liposomes. *In vitro* shelf-life stability studies demonstrated that the product was stable for at least one year.

### Animal Studies

Dogs presenting to the local institutions with histologically or cytologically confirmed masses of any histology and any stage were eligible for entry. The diagnosis was made by ultrasound-guided tru-cut histologic or 20-gauge needle-aspirate cytology. The dogs that had necropsy also underwent histologic confirmation of the imaged lesions. A sub-set of dogs went to surgery after the final imaging session, and all grossly abnormal tissues identified either intraoperatively or on CT scans, were excised, when feasible, or biopsied. As the study progressed, preference was given to cases that had a prior history or a diagnosis of primary or metastatic liver tumors. Dogs were required to have adequate organ function as indicated by complete blood count and serum biochemistry profile. Dogs were also required to have a veterinary comparative oncology group (VCOG) general performance grade of 0 (fully active, able to perform at pre-disease level) or 1 (mild lethargy over baseline; diminished activity from pre-disease level, but able to function as an acceptable pet) [18]. None of the dogs that participated in this study were severely compromised. During the administration of contrast agent, the dogs were monitored for heart rate, respiration rate and blood pressure. Physical examinations were conducted at follow-up.

### Liposomal-I Contrast Administration Protocol

Dogs were administered Liposomal-I at an iodine dose of 275 mg/kg body weight (2.5 mL/kg volume dose). The iodine dose used in this study is similar to previous studies, performed in small animal models, that have demonstrated visibility of solid tumors [19,20]. The contrast agent was administered to anesthetized dogs immediately following their pre-contrast baseline CT scan. Since dogs are hyper-sensitive to liposome-based infusion reactions, a multi-step infusion protocol was instituted for the administration of liposomal contrast agent. Administration of was performed as a slow intravenous infusion using an infusion pump at a rate of 0.2 mL/min for the first 5 minutes of the infusion period. In the absence of significant alterations in blood pressure (BP), heart rate (HR), and other signs of adverse effects, the dose rate was escalated over a 1- to 2-minute period to an infusion rate of 2 mL/min, which was continued for 5 minutes and then increased to 5 mL/min for the remainder of the infusion. In dogs experiencing adverse events, infusion was slowed or transiently discontinued and intravenous fluid therapy continued prior to continuation of infusion.

### CT Imaging

Animals were transported from the veterinary center to the diagnostic imaging department. In all dogs, a light plane of general anesthesia was induced with propofol (4–6 mg/kg IV) and maintained with isoflurane inhalational anesthesia under the direction of the anesthesia services at each institution.

Imaging studies were performed on 16-slice and 64-slice MDCT scanners (Aquilion-16, Toshiba Medical Systems, Tustin, CA; Discovery CT750HD, GE Healthcare, Little Chalfont, UK). The dogs were divided into two groups. The dogs in Group 1 (N = 9) were imaged using Liposomal-I only. Group 2 dogs (N = 4) were imaged using a conventional contrast agent, Iohexol, and then using Liposomal-I, each sub-study separated by at least 90 minutes. In seven of the nine Group 1 dogs, a pre-contrast baseline scan was acquired, followed by administration of Liposomal-I. Post-contrast scans were acquired at 15 minutes and 24 hours after completion of Liposomal-I administration. The remaining two dogs received only a post-24 hour scan. These two dogs were awake during administration of the Liposomal-I contrast agent.

A comparative imaging study was conducted in the four Group 2 dogs. Following induction of anesthesia, each dog underwent a pre-contrast baseline scan followed by a triple-phase conventional CECT scan. Conventional CECT scans were acquired as per standard in-house protocol. Briefly, each dog received a bolus intravenous injection of Iohexol (Omnipaque™ - 300 mg I/mL, GE Healthcare, Little Chalfont, UK,) at a dose of 2.2 mL/kg (660 mg I/kg) at a rate of 5 mL/s. A ninety-minute washout period was observed after completion of conventional CECT to facilitate systemic clearance of Iohexol. Thereafter, Liposomal-I was administered and post- contrast scans were acquired at 15 minutes and 24 hours after completion of Liposomal-I administration.

Pre-contrast and Liposomal-I-enhanced CT scans were acquired at 120 kVp with the following settings: tube current: 100–400 mA; slice thickness: 0.625–1.25 mm. All images were reconstructed using a standard kernel available on the CT scanner.

### Image and Data Analysis

A veterinary radiologist reviewed the CT images (A.F.S, 15 years experience). In Group 1 dogs, quantitative analysis of CT images was performed in Osirix (version 5.8.5 64-bit; Pixmeo, Bernex, Switzerland) (Z.A.S, 4 years experience). Circular regions of interest were drawn in major blood vessels (descending aorta, inferior vena cava and portal vein), liver, spleen, kidney cortex, bladder and muscle (erector spinae). For each organ/blood vessel, three ROIs were drawn on different images. Results were presented as mean CT signal (in Hounsfield Units, HU) with standard deviation. Mean vascular CT signal was determined as the average of CT signal in the descending aorta, inferior vena cava and portal vein.

### Statistical Analysis

A Kruskal—Wallis test was used for statistical analysis of signal enhancement in different organs. A paired t-test was applied for pairwise group comparison of signal enhancement between different time points. P values < 0.05 adjusted with Bonferroni correction were considered to indicate a statistically significant difference.

## Results

**Fig 1:**
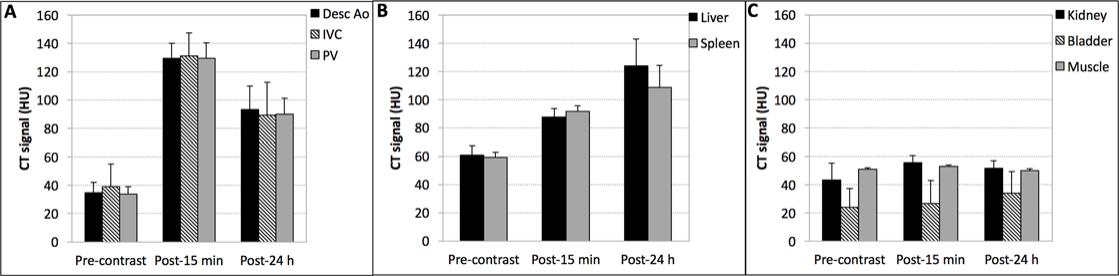
Signal attenuation properties of Liposomal-I. (A) Vascular CT signal was determined in descending aorta (Desc Ao), inferior vena cava (IVC) and portal vein (PV). (B) CT signal was determined in the liver and spleen, primary organs for systemic clearance of Liposomal-I, (C) CT signal was also measured in the kidney (cortex), bladder and muscle (erector spinae). CT signal measurements were determined in images acquired at 120 kVp. Measurements were performed pre-contrast and then at 15 min and 24 h after administration of Liposomal-I. The dose of Liposomal-I administered was 275 mg I/kg. Significant differences were observed in CT signal measured at each time point for all the ROIs in Fig 1A and Fig 1B (p < 0.05). In Fig 1A, CT signal measured for different vascular structures at each time were not significantly different. In Fig 1C, the CT signal in kidney, bladder and muscle measured at each time point were not significantly different (p > 0.05).

A total of 13 dogs were recruited into the study. Mean age was 10.1 years (range 4–15 years) and mean body weight was 30.2 kg (range 23.1–36.6 kg). The diagnoses of all the cases are provided in Table 1. The administration of Liposomal-I resulted in statistically significant (p < 0.05) and uniform signal enhancement in the arterial and venous system (Fig 1A). Mean vascular CT signal increased from ~ 36 HU at pre-contrast to ~ 130 HU at 15 minutes post-Liposomal-I administration, followed by a gradual decrease to ~ 91 HU at the 24 hour period. The gradual decay in vascular CT signal paralleled the rise of CT signal in the liver and spleen, the primary organs for systemic clearance of liposomal agents (Fig 1B). The absence of statistically significant signal enhancement (p < 0.05) in the kidney (cortex) and bladder exemplified the *in vivo* stability of the long circulating agent and its non-renal systemic clearance (Fig 1C). In the liver, the hepatic vasculature appeared hyper-enhanced relative to the liver parenchyma at the post-15 min scan (S1A Fig). In the post-24 h scan, the hepatic vasculature appeared iso- to hypo-enhanced due to relatively high CT signal in the liver parenchyma. The homogenous signal enhancement of the liver parenchyma, both in the post-15 min and post-24 h images, appeared to be an imaging characteristic of normal liver uptake of Liposomal-I.

**Table 1:** Case information of dogs imaged in the study.

ID	Breed	Age	Weight (kg)	Diagnosis (Histology/Cytology)
**Group 1**: Imaged with Liposomal-I only				
1	Golden Retriever	4y 2m	37	Anaplastic bone sarcoma of right distal radius; anaplastic sarcoma metastasis to lungs
2	Pit Bull	15y	27	Complex ductular carcinoma in first left mammary gland; splenic hemangiosarcoma; grade 3 mast cell tumor in right thoracic dermis
3	Labrador Retriever	9y 4m	30	Sarcoma in right femur; pulmonary mass: histiocytic sarcoma
4	Golden Retriever	12y	33	Well-differentiated hepatocellular carcinoma
5	LabX	9y	32	Splenic hematoma; moderate portal fibrosis, vacuolization and inflammation; jejunal lymph node hyperplasia
6	LabX	10y 10m	28	Left peri-renal hemangiosarcoma; splenic nodular hyperplasia; liver copper deposition
7	Boxer	8y	36	Splenic hematoma; liver vacuolization and lipogranulomas
8	Mix	8y 6m	27	Splenic hemangiosarcoma; liver and omentum metastasis
9	Golden Retriever	10y 10m	30	Grade 2 splenic fibrohistiocytic nodule
**Group 2**: Imaged with Iohexol and then with Liposomal-I, each imaging separated by atleast 90 minutes				
10	German Shepherd	8y 7m	32	Hepatocellular carcinoma
11	Golden Retriever	9y 2m	27	Splenic hemangiosarcoma; hepatic hemangiosarcoma
12	Basset Hound	14y 8m	23	Retroperitoneal mass: neuroendocrine carcinoma, likely paraganglioma; pulmonary mass: histiocytic sarcoma; splenic mass (large): hematoma with lymphoid hyperplasia with extramedullary hematopoiesis; splenic mass (small): myelolipoma
13	LabX	10y 8m	30	Hepatocellular carcinoma

**Fig 2:**
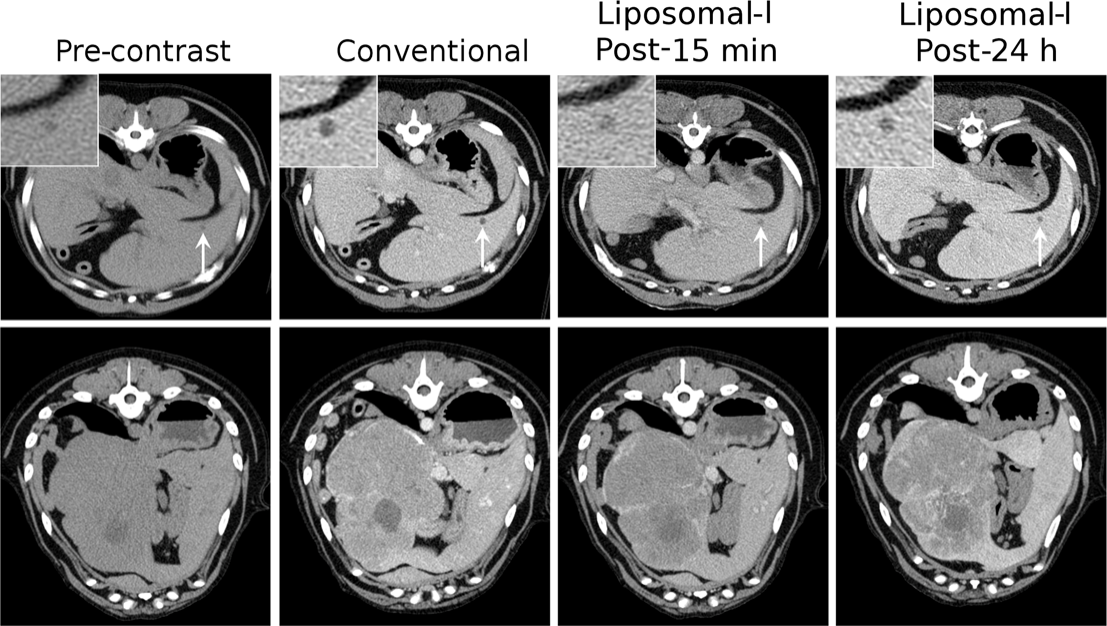
Signal enhancement pattern of Liposomal-I in primary liver tumors. Axial CT images demonstrating the effect of imaging time point on visualization of hepatocellular carcinoma in images acquired post-administration of 275 mg I/kg Liposomal-I. Images are presented for a small (0.5 cm, top row, white arrows) and a large (12.5 cm, bottom row) case of HCC. For comparison, delayed images were also acquired using a conventional contrast agent, Iohexol at 660 mg I/kg. A 90 min washout period was observed between the conventional CECT and administration of Liposomal-I. (WL/WW: 40/350).

**Fig 3:**
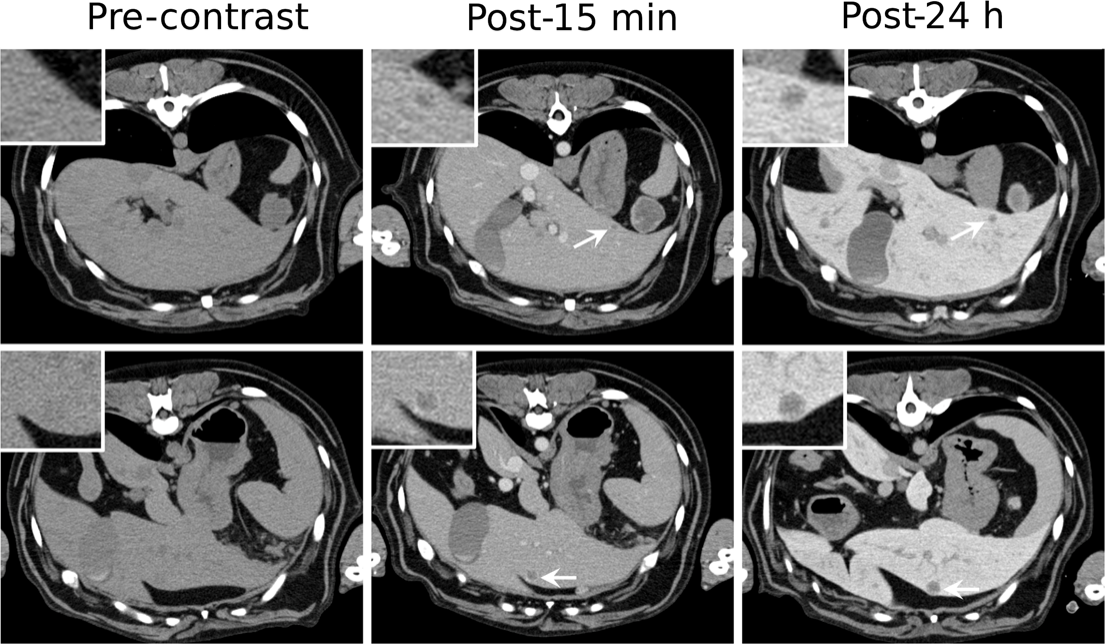
Signal enhancement pattern of Liposomal-I in metastatic liver tumors. Axial CT images demonstrating the effect of post-Liposomal-I imaging time point on visualization of metastatic liver hemangiosarcoma (white arrows). The 0.5 cm (top row) and 1 cm (bottom row) lesions are better visualized on the post-24 hour images due to increased liver uptake of the contrast agent. (WL/WW: 40/350).

Several tumors types were investigated in the current study (Table 1). A few cases of tumor types visualized using Liposomal-I are presented below. Representative CT images for other tumor types, as well as non-tumor pathologies encountered in the study (splenic hematoma, splenic nodular hyperplasia and myelolipoma) are presented in the S1 Fig. Liposomal-I enabled visualization of small and large primary liver tumors (Fig 2). Small, sub-cm sized hepatocellular carcinoma (HCC) lesions grossly appeared as hypo-enhanced, relative to adjacent parenchyma, in post-15 min and post-24 h Liposomal-I CECT images. In comparison, small HCC tumors also appeared hypo-enhanced in the iohexol CECT images. Large HCC tumors showed a heterogeneous intra-tumoral pattern of signal enhancement on the Liposomal-I CECT images. Intra-tumoral signal enhancement appeared more intense in the post-24 h Liposomal-I images than in the post-15 min images. In contrast, the normal liver parenchyma demonstrated a homogeneous pattern of signal enhancement. Liposomal-I also enabled visualization of metastatic liver tumors (Fig 3). The metastatic lesions appeared as hypo-enhanced relative to the surrounding normal parenchyma. Lesions as small as 5 mm were visible in scans acquired at 15 min and 24 h after Liposomal-I administration. The lesions become more conspicuous in the post-24 h images due to higher contrast between the lesion and the surrounding normal parenchyma. Compared to tumors, hematomas showed a markedly different enhancement pattern with regions of very high signal intensity, at the 24 h time point (S1B Fig).

**Fig 4:**
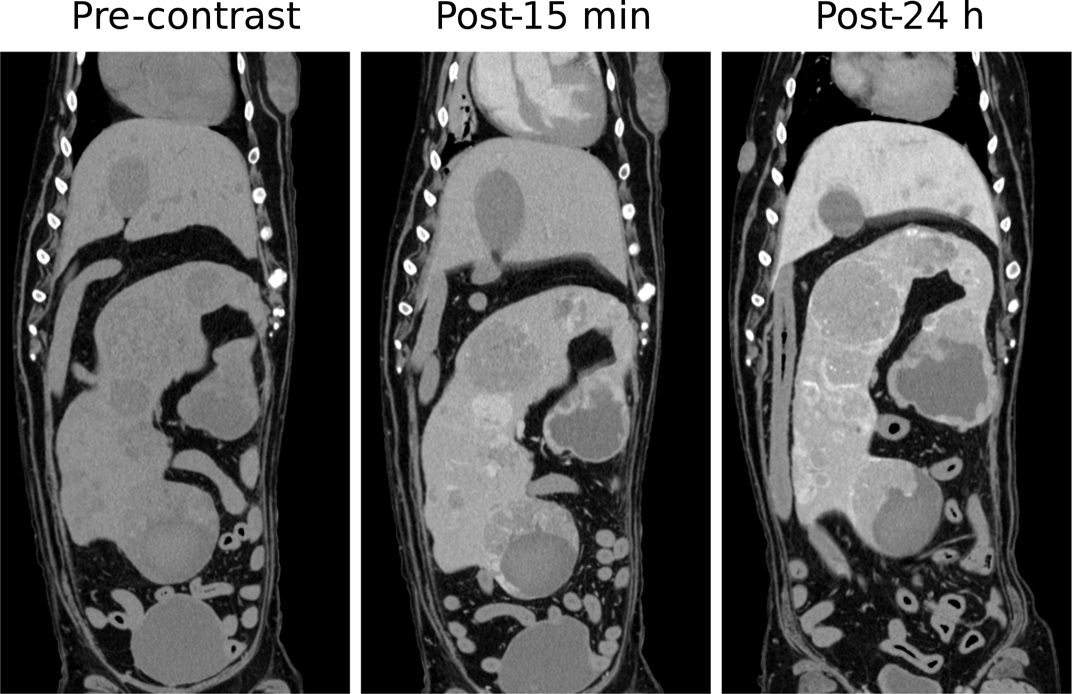
Imaging of splenic hemangiosarcoma. Coronal images demonstrating changes in the CT signal and enhancement pattern in splenic hemangiosarcoma at 15 minutes and 24 hours after administration of Liposomal-I. (WL/WW: 40/350).

**Fig 5:**
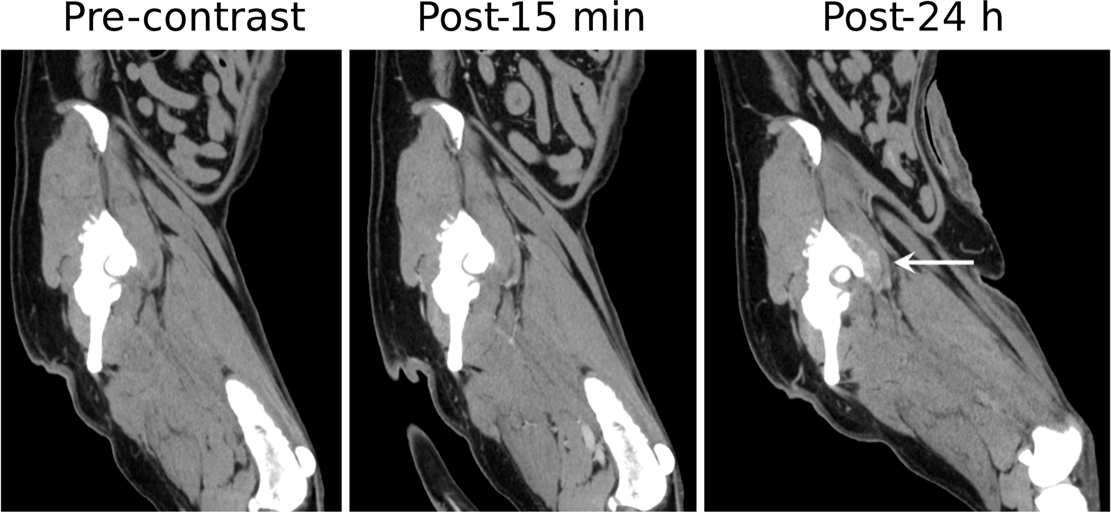
Imaging of histiocytic sarcoma. Sagittal images demonstrating changes in CT signal and enhancement pattern in a histiocytic sarcoma (white arrow) before and after administration of Liposomal-I. Soft tissue enhancement adjacent to the acetabulum is visible on the post-24 hour image only (arrow). (WL/WW: 40/350).

**Fig 6:**
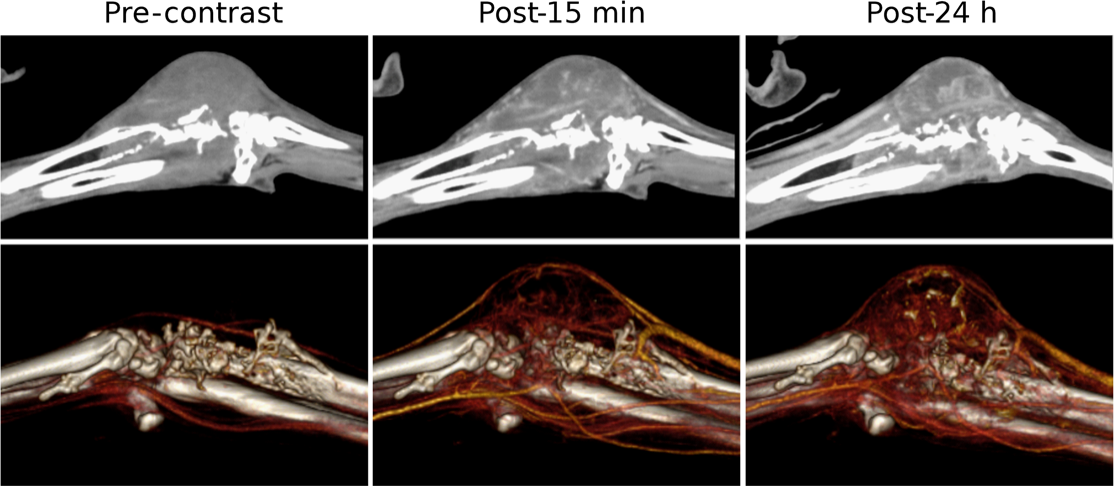
Imaging of anaplastic bone sarcoma. Sagittal cross-sectional (top row) and 3D volume-rendered (bottom row) images demonstrating changes in CT signal and enhancement pattern in a right distal radial anaplastic bone sarcoma. The post-24 h images show a higher region of signal enhancement indicating accumulation of Liposomal-I within the tumor tissue. (WL/WW: 40/350).

**Fig 7:**
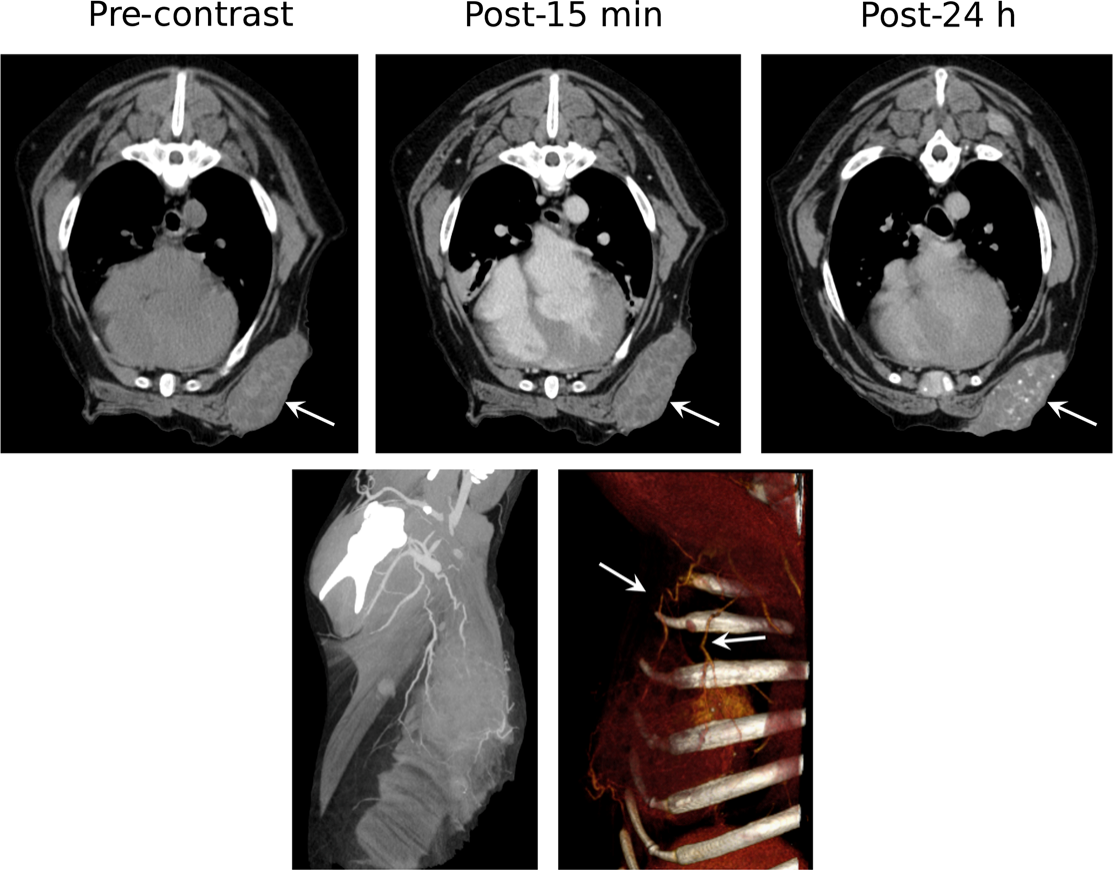
Imaging of complex ductular carcinoma. Top row–Axial images demonstrating changes in CT signal and enhancement pattern in a complex ductular carcinoma (white arrow). Distinct focal spots of hyper-intense CT signal are demonstrated within the tumor tissue in the post-24 h image. Bottom row–Maximum intensity projection (MIP) image and 3D volume-rendered image, generated from the post-15 min images, demonstrating the tumor-associated tortuous vessels. (WL/WW: 40/350).

Splenic hemangiosarcomas, an aggressive tumor type commonly diagnosed at an advanced-stage in dogs, were also visualized using Liposomal-I (Fig 4). The lesions had a similar appearance in the post-15 min and post-24 h images, demonstrating a heterogeneous signal enhancement pattern. Large intra-tumoral regions, suggestive of avascularized tissue, appeared as hypo-enhanced at both the time points in the post-Liposomal-I scans. The post-24 h Liposomal-I scan demonstrated higher variations in intra-tumoral signal intensity compared to the post-15 min scan, indicative of multi-focal pathology and spatial variability in tumor blood vessel permeability to the liposomal contrast agent. Two of the dogs presented with splenic nodular hyperplasia (S1C Fig). The lesion appeared hypo-enhanced in the post-15 min scan and post-24h scan. Interestingly, a circular rim of high CT signal was observed in the post-24h images. Splenic myelolipoma, observed in one of the dogs, showed a similar signal enhancement pattern to that of splenic nodular hyperplasia (S1D Fig). Tumors of non-RES organs showed a different mode of lesion visibility. Histiocytic sarcoma demonstrated intra-tumoral signal enhancement in the post-24 h Liposomal-I scan only (Fig 5). No evidence of signal enhancement was observed, in the target region, in the post-15 min Liposomal-I scan. A similar temporal pattern of signal enhancement was observed in a dog presenting with anaplastic bone sarcoma in the distal radius (Fig 6). Liposomal-I also enabled visualization of complex ductular carcinoma (Fig 7). A heterogeneous signal enhancement pattern was observed in the post-15 min Liposomal-I images. Tumor associated vessels were also visible in the post-15 min scan. Multi-focal spots of intense signal enhancement were observed in the post-24 h Liposomal-I scan, indicative of permeable intra-tumoral vasculature. Several of the dogs presented with metastatic lung masses. Similar to the other non-RES tumors, the lung lesions showed multi-focal spots of signal enhancement (S1E Fig).

Nine out of thirteen dogs experienced acute infusion reactions, with clinically measurable changes observed immediately at the onset of Liposomal-I administration. The reactions typically lasted for 5–10 min with physiological manifestations of the reactions consisting of a mild to moderate transient drop in blood pressure and a concomitant increase in the heart rate (S1F Fig). Two of the dogs, that only had the post-24 h Liposomal-I scan, were awake during the administration of the contrast agent. No observations of nausea or vomiting were noted in these dogs. No other adverse events attributed to the administration of Liposomal-I were seen in any of the dogs. The majority of the dogs returned home following CT and surgery. Although not part of the current study, a subset of dogs (N = 4) underwent clinical blood chemistry (CBC) and biochemistry analysis at 1 week to 2 months after Liposomal-I administration with no significant findings.

## Discussion

Studies have shown that dogs and humans share several characteristics of cancer initiation and progression [21]. The use of companion dogs, therefore, enables investigation in natural spontaneously occurring tumors at clinically-relevant lesion sizes on a whole-body CT scanner with hardware setup and scan protocols that are routinely used in clinical practice. In this feasibility study, the imaging characteristics of Liposomal-I were investigated in companion dogs with spontaneously occurring tumors. The Liposomal-I enabled visualization of a variety of malignant lesions with signal enhancement patterns that varied depending on lesion size and location. Furthermore, the long circulating property of Liposomal-I resulted in uniform and stable signal enhancement in the vascular system with a gradual systemic clearance of the agent via the liver and spleen.

CT imaging was performed at 15 minutes and 24 hours post-Liposomal-I administration to determine signal attenuation during early-phase and delayed-phase of the agent’s blood half-life. While vascular CT signal during early-phase imaging was dependent on administered iodine dose and circulating blood volume, during delayed-phase imaging, it was dependent on the Liposomal-I RES uptake rate and differential extravasation and accumulation in tumor tissue. The vascular CT signal in post-15 min images, however, was lower compared to a conventional CECT scan. The lower CT signal is a consequence of smaller iodine dose administered as Liposomal-I (275 mg I/kg) when compared with a conventional CECT (660 mg I/kg) and dilution of Liposomal-I in the vascular compartment during steady-state imaging. In the liver, hepatic vessels appeared hyper-enhanced in the post-15 min images, whereas, they appeared iso- to hypo-enhanced in the post-24 h images. The variability in the contrast between hepatic vasculature and liver parenchyma in the post-24 h images can be attributed to inter-animal differences in RES uptake of Liposomal-I.

Unlike conventional contrast agent, which has rapid wash-in/wash-out tumor kinetics, liposomes gradually extravasate, through the permeable tumor vasculature, and accumulate in tumors, a phenomenon known as enhanced permeation and retention (EPR) [22]. Intra-tumoral transport of liposomes primarily occurs by a restricted convective process and further hindered by high interstitial fluid pressures [23,24]. The signal enhancement patterns of tumor lesions varied depending on their location and size. In the liver, large primary tumors appeared hypo-enhanced compared to normal liver parenchyma during early-phase imaging. In delayed-phase scans, the lesions were visible due to heterogeneous signal enhancement pattern within the tumor; on the contrary, normal liver was homogenously opacified. Since Liposomal-I is primarily distributed in the blood compartment, the heterogeneous pattern of signal enhancement in the post-15 min scan infers to spatial variability in the tumor perfusion. During delayed-phase imaging, the heterogeneous enhancement pattern is likely a consequence of regional variation in tumor vessel permeability. Furthermore, intra-tumoral signal enhancement appeared more intense in post-24 h images than in post-15 min images, a characteristic that can be attributed to the extravasation and intra-tumoral retention of liposomal particles. Small malignant lesions (< 1 cm) appeared hypo-enhanced during early-phase imaging, likely due to perfusion differences between tumor tissue and normal parenchyma. These lesions remained hypo-enhanced in the delayed-phase imaging; however, lesion conspicuity was improved due to increased contrast between normal parenchyma and tumor tissue. Although the small lesion size precluded intra-tumoral assessment of signal enhancement, retention of liposomal contrast agent within the metastatic lesion cannot be ruled out [25,26]. Evaluation of lesion visibility could not be studied beyond 24 hours due to protocol constraints; however, the lesions are likely going to be visible for several days due to the slow clearance of Liposomal-I from tumors and RES organs. Prolonged visualization of lesions could greatly aid CT image-guided biopsy and treatment of tumors, especially for liver tumors that have poor opacification characteristics. For cancer imaging, the use of Liposomal-I eliminates the variability surrounding bolus rate and optimal scan timing, resulting in visualization of lesions strictly based on their inherent architecture.

Liposome-based CT contrast agents have been investigated for clinical translation [10,27]. However, the choice of lipid composition (use of charged lipids), large particle size and the rapid systemic clearance (due to absence of PEG polymer coating and/or large particle size) presented safety concerns. In addition, several other promising nanoparticle-based CT agents are currently under pre-clinical development [14,28–31]. Although the liposomal-I used in this study addresses some of the above limitations, detailed safety and toxicology studies are required to understand the risks factors and the potential for clinical translation of this agent. Recently, Hansen et al. evaluated the utility of Cu-64 liposomal PET contrast agent to probe the EPR effect, for monitoring of intra-tumoral liposome accumulation in dogs with spontaneous cancer [32]. The liposomal-iodine contrast agent described here, combined with the high spatial-resolution of CT imaging, could serve a similar purpose; however the contrast sensitivity of a CT agent is likely to be poorer than its PET counterpart. On-going advances in CT scanners are likely to increase contrast sensitivity, however, such methodologies will require further investigation [33,34].

A slow infusion protocol was used for the administration of the liposomal-I to minimize the severity of acute infusion reactions. A variety of exogenous compounds, including liposomes and conventional ionic contrast agents, are known to elicit acute infusion reactions [35–37]. The acute infusion reactions are manifested due to activation of the complement system, resulting in transient cardiopulmonary changes, a phenomenon known as complement activation related pseudoallergy (CARPA) [36]. These reactions have been extensively studied in a variety of species including dogs, which are known to be hyper-sensitive, and approaches to mitigate these reactions have been investigated, including the use of a slow initial infusion rate, pre-medications using corticosteriods, anti-histamines, anti-C5a antibodies and placebo liposomes [37,38]. Pre-clinical studies, performed in clinically-relevant large animal models, to minimize the incidence and severity of infusion reactions are needed since the risk of infusion reactions, especially in seriously ill patients (both canine and humans), such as those with cardiopulmonary distress, can pose major risks. Furthermore, the long infusion protocol can also present challenges in human clinical translation due to substantial changes in workflow process.

The study has limitations. First, the small cohort size of the study precluded quantitative analysis of lesion imaging. Second, the effect of Liposomal-I dose on lesion visibility as a function of imaging time, for different tumor types and lesion sizes as well as non-malignant lesions, needs to be studied. Third, an understanding of the Liposomal-I clearance rate from tumor and RES organs needs to be studied for applications in gastrointestinal imaging. While the current work provides preliminary efficacy data on the agent’s imaging characteristics in companion dogs with naturally occurring cancer, future studies addressing the above questions are warranted to identify the target population (cancer type), in both canine and human cancer patients, wherein the utility of Liposomal-I would prove beneficial over conventional iodine agents.

## Supporting Information

S1 Fig Representative CT images for other tumor types, as well as non-tumor pathologies encountered in the study. Signal enhancement in normal liver **(S1A Fig)**. Signal enhancement pattern of Liposomal-I in a splenic hematoma with extramedullary hematopoiesis and lymphoid hyperplasia **(S1B Fig)**. Signal enhancement pattern of Liposomal-I in splenic nodular hyperplasia **(S1C Fig)**. Signal enhancement pattern of Liposomal-I in a splenic myelolipoma **(S1D Fig)**. Signal enhancement pattern of Liposomal-I in metastatic lung tumors **(S1E Fig)**. Acute infusion reaction-induced cardiovascular changes **(S1F Fig)**.
(PDF)
