# Supplementary material for: Computed Tomography Imaging of Solid Tumors Using a Liposomal-Iodine Contrast Agent in Companion Dogs with Naturally Occurring Cancer
Source: PLoS One. 2016 Mar 31;11(3):e0152718. doi: 10.1371/journal.pone.0152718 (PMC4816501; doi:10.1371/journal.pone.0152718)
Supplement: S1 Fig — Signal enhancement in normal liver (S1A Fig). Signal enhancement pattern of Liposomal-I in a splenic hematoma with extramedullary hematopoiesis and lymphoid hyperplasia (S1B Fig). Signal enhancement pattern of Liposomal-I in splenic nodular hyperplasia (S1C Fig). Signal enhancement pattern of Liposomal-I in a splenic myelolipoma (S1D Fig). Signal enhancement pattern of Liposomal-I in metastatic lung tumors (S1E Fig). Acute infusion reaction-induced cardiovascular changes (S1F Fig). (PDF) [file pone.0152718.s001.pdf]

Computed Tomography Imaging of Solid Tumors Using a Liposomal-Iodine Contrast Agent in Companion Dogs With Naturally Occurring Cancer

Supporting Information

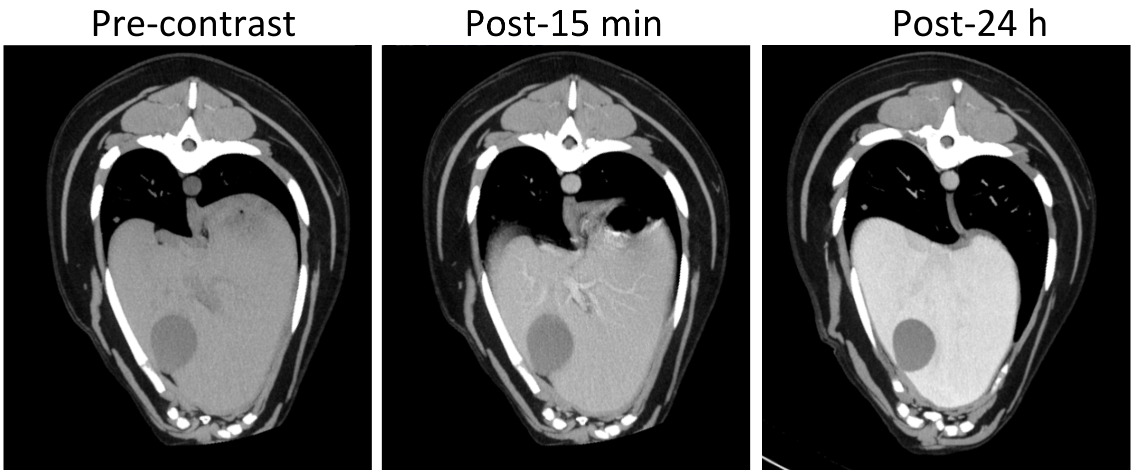

S1A Fig. Signal enhancement in normal liver. Axial CT images demonstrating temporal changes in signal enhancement patterns in liver parenchyma and hepatic vasculature. Images were acquired pre-contrast and at 15 minutes and 24 hours after administration of Liposomal-I (275 mg I/kg). Images were acquired at 120 kVp. (WL/WW: 40/350)

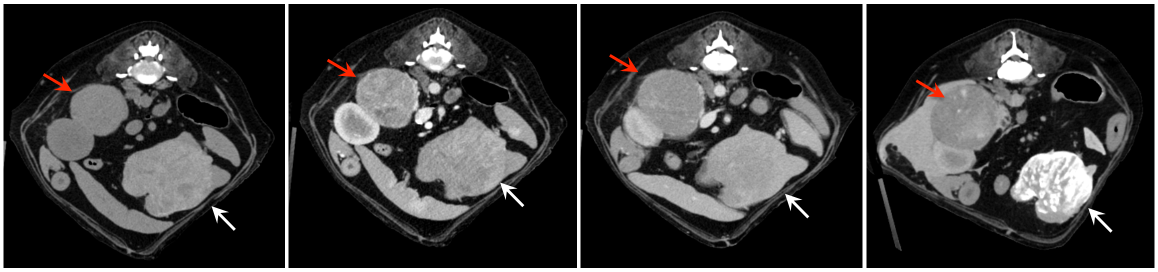

S1B Fig. Signal enhancement pattern of Liposomal-I in a splenic hematoma with extramedullary hematopoiesis and lymphoid hyperplasia. Axial CT images demonstrating the effect of post- Liposomal-I imaging time point on visualization of splenic hematoma (white arrow). Note the intense uptake on the post 24 h scan. The retroperitoneal neuroendocrine carcinoma is also visible in the images (red arrow). For comparison, images were also acquired using a conventional contrast agent, Iohexol. The iodine dose for conventional contrast agent and Liposomal-I were 660 mg I/kg and 275 mg I/kg, respectively. (WL/WW: 40/350)

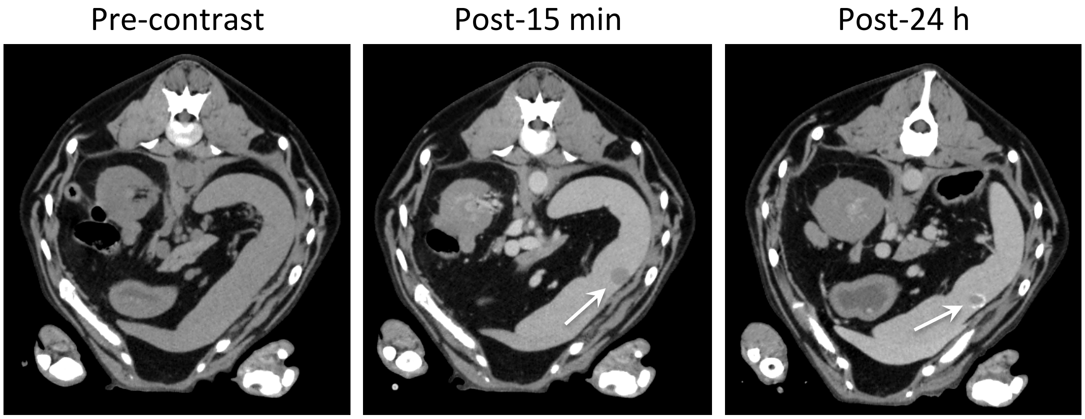

S1C Fig. Signal enhancement pattern of Liposomal-I in splenic nodular hyperplasia. Axial CT images demonstrating the enhancement pattern in splenic nodular hyperplasia before and after administration of 275 mg I/kg of Liposomal-I. (WL/WW: 40/350)

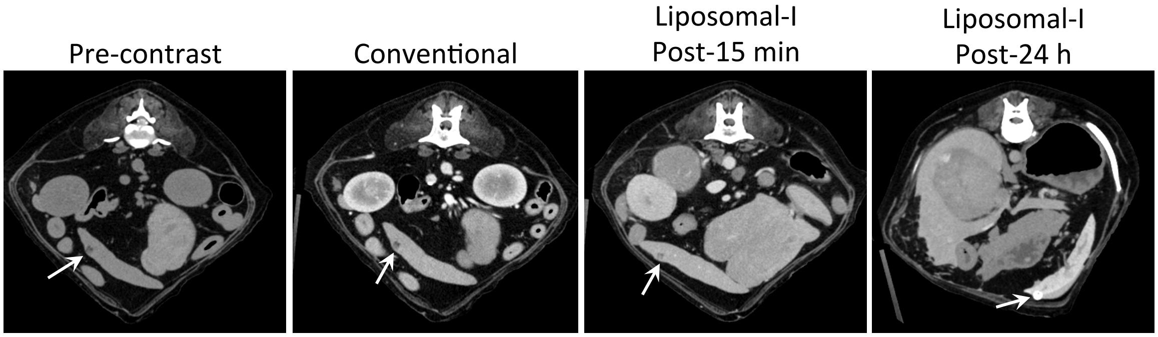

S1D Fig. Signal enhancement pattern of Liposomal-I in a splenic myelolipoma. Axial images demonstrating visualization of a splenic myelolipoma on conventional CECT and Liposomal-I CECT. The iodine dose for conventional contrast agent and Liposomal-I were 660 mg I/kg and 275 mg I/kg, respectively. (WL/WW: 40/350)

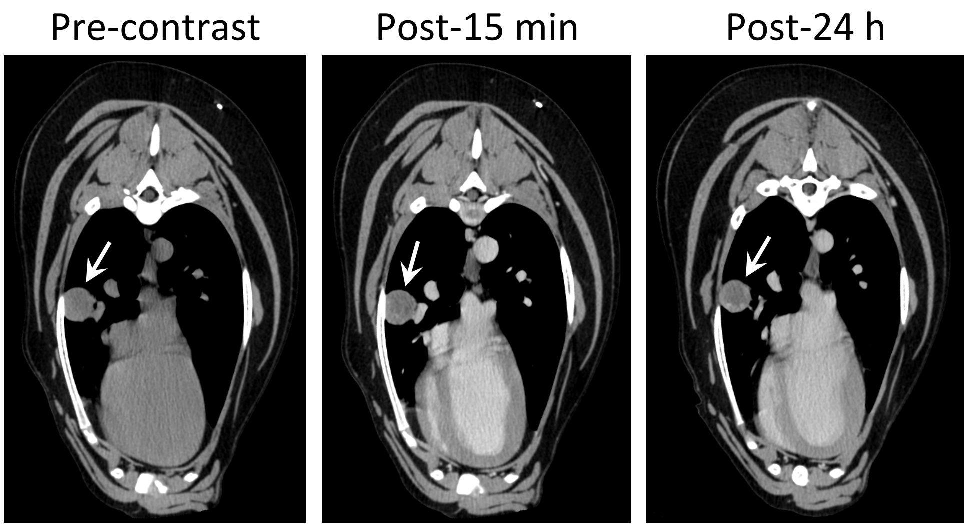

S1E Fig. Signal enhancement pattern of Liposomal-I in metastatic lung tumors. Axial CT images obtained from a dog with metastatic lung lesions from a radial osteosarcoma. (WL/WW: 40/350)

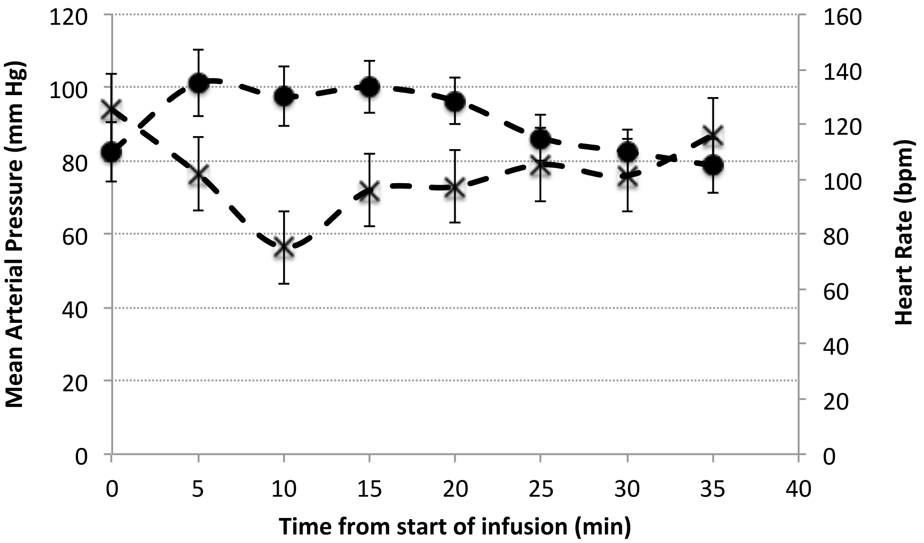

S1F Fig. Acute infusion reaction-induced cardiovascular changes. Effect of Liposomal-I administration on changes in mean arterial pressure (mm Hg) and heart rate (beats per min, bpm). Values are presented as mean and standard errors. '**X**' denote mean arterial pressure; '●' denote heart rate.
